# Oocyte cryopreservation for fertility preservation in transgender and gender diverse individuals: a SWOT analysis

**DOI:** 10.1007/s10815-025-03579-2

**Published:** 2025-07-28

**Authors:** Julia E. Barbe, Federico Del Vento, Dehlia Moussaoui, Victoria L. Crofts, Michal Yaron, Isabelle Streuli

**Affiliations:** 1 Division of Gynecology, Department of Woman, Child and Adolescent Medicine, Faculty of Medicine of Geneva, Geneva University Hospitals, Geneva, 1205 Switzerland; 2 Division of General Pediatrics, Department of Woman, Child and Adolescent Medicine, Geneva University Hospitals, Geneva, Switzerland

**Keywords:** Fertility preservation, Transgender and gender diverse individuals, Oocyte cryopreservation, Transgender health, SWOT analysis

## Abstract

Gender affirming hormone therapy may be used to relieve dysphoria among transgender or gender-diverse individuals. However, the long-term effects of these treatments on reproductive health remain uncertain. Before initiating hormone therapy, it is essential that patients are fully informed about the potential impact on their reproductive capabilities. Fertility preservation options, such as oocyte cryopreservation, should be offered to gender diverse individuals who were assigned female at birth. To assist healthcare providers in navigating the complexities of fertility preservation for these patients, we conducted a SWOT (Strengths, Weaknesses, Opportunities, and Threats) analysis focused of oocyte cryopreservation. This analysis aims to ensure that decisions are both clinically sound and aligned with the patient's reproductive goals.

## Background

Transgender and gender diverse individuals (TGD) have a gender identity that differs from their sex assigned at birth. They may suffer from gender dysphoria, which is the psychological distress caused by the discrepancy between the sex assigned at birth and the individual’s gender [1]. The prevalence of gender incongruence in the population is difficult to determine but estimated between 0.5 and 1.3% [2, 3]. To relieve gender dysphoria, some individuals may seek gender affirming treatments (GAT) such as gender affirming hormone therapy (GAHT) and surgery (GAS) as well as psychological and social support. The choice of hormonal treatment depends on the pubertal stage of the individual and current guidelines [4]. GnRH agonists reversibly block puberty from Tanner stage 2 [4] and, consequently, the development of unwanted and distressing secondary sexual characteristics. After this, most TGD individuals assigned female at birth opt for gender affirming hormonal treatments by initiating testosterone therapy to develop secondary sexual characteristics compatible with gender identity [5]. Furthermore, some TGD individuals may choose to undergo gender-affirming procedures, such as hysterectomy and/or oophorectomy, which consequently result in sterilization. Although these treatments may reduce dysphoria and improve quality of life [6, 7], they have an impact on future reproductive capacity [9, 10]. Therefore, the World Professional Association for Transgender Health (WPATH), American Society for Reproductive Medicine (ASRM), and European Society of Human Reproduction and Embryology (ESHRE) recommend that health professionals address the potential effect of gender affirming treatments on fertility and counsel on fertility preservation options before initiating treatment [2, 11, 12].

Despite the widespread availability of assisted reproductive techniques (ART) and recommendations by expert societies, several studies have demonstrated that less than 5% of TGD individuals pursue fertility preservation (FP) when initiating GAT [13–16]. Notably, some research indicate that FP rates are even lower among TGD individuals assigned female at birth compared to those assigned male at birth [16].

**Fig. 1:**
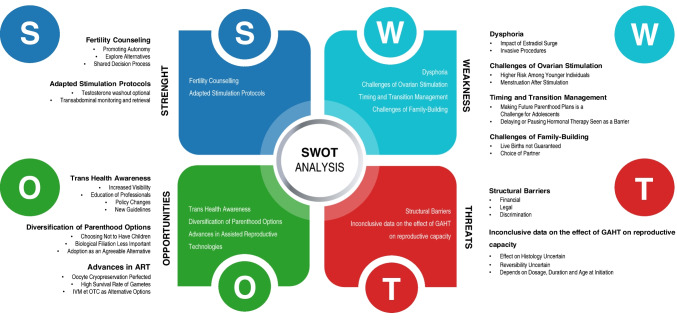
SWOT analysis of fertility preservation in transmasculine people. SWOT—an acronym of Strengths, Weaknesses, Opportunities, and Threats—is a structured planning method that evaluates the internal and external factors that are favorable or not in a project. Applied to clinical problems, SWOT gives a thorough analysis of a given clinical question and can help decision making and strategic planning

To improve our counseling of TGD patients prior to initiating GAHT, which may adversely affect reproductive capacity, we used a comprehensive SWOT analysis of oocyte cryopreservation for fertility preservation in TGD individuals assigned female at birth. SWOT stands for Strength, Weakness, Opportunities, and Threats. It is a structured planning tool used to evaluate both internal and external factors influencing a project. When applied to clinical decision-making, internal factors are those that directly affect the patient. External factors encompass broader societal and medical considerations. To make the analysis more intuitive, we are changing the order to emphasize the broader societal and medical factors first, providing a contextual framework before delving into specific internal issues. This approach aims to clarify the advantages and drawbacks of fertility preservation for trans men by integrating both external and internal factors effectively (Fig. 1).

## Threats

### Structural barriers and discrimination

Despite WPATH providing guidelines for health professionals, there is no standardized protocol for fertility counseling, and comprehensive information on FP for families and individuals undergoing GAT is limited. FP is often divided into medical and non-medical categories. Medical FP typically applies to conditions like cancer or endometriosis, where fertility preservation is recommended prior to gonadotoxic treatments, and these cases are often more likely to be covered by insurance [18, 19]. In contrast, social FP (e.g., for delaying family planning) is categorized as non-medical, with limited or no coverage [20, 21]. This distinction creates uncertainty for TGD individuals, as there is no consensus on how FP is categorized in this context, despite the financial burden posed by the costs of gamete retrieval, storage, and future in vitro fertilization (IVF) procedures [13, 14, 22, 23]. In regions with restrictions on storage duration, FP categorized as medical may qualify for extensions, further highlighting the impact of classification on access and affordability [25].

Discrimination within healthcare settings compounds these structural barriers. TGD individuals frequently experience stigma, a lack of knowledgeable providers, and limited support, which can lead to mistrust and avoidance of healthcare services [26, 27]. Many TGD individuals also face self-stigma when considering family planning, fearing discrimination for themselves and their future children. For example, a survey in Belgium found that 38% of TGD individuals who wanted to become parents feared discrimination against their child, while 32.6% feared discrimination against themselves as parents [22]. TGD individuals may also face stigma and discrimination from adoption agencies and social workers, discouraging many from pursuing adoption or fostering [28].

### Inconclusive data on the effect of GAHT on reproductive capacity

There is a lack of conclusive data regarding the long-term effect of testosterone on reproductive capacity, as impact may vary depending on dosage, serum levels, duration of use, and age at GAHT initiation. Early research suggested that testosterone could induce a PCOS-like effect on ovaries [29–31] or cause ovarian cortex and stroma hyperplasia [33], though the reversibility of these changes remains unknown. Additionally, studies found that TGD individuals had a higher yield of mature oocytes collected after stimulation, possibly due to testosterone creating a PCOS-like environment that enhances follicular maturation [34, 35]. Current research suggests that there are no significant histopathological differences in ovarian tissue after testosterone therapy [31, 37, 38]. Furthermore, emerging data show that many TGD individuals can achieve pregnancy or successfully use their oocytes to initiate pregnancy, even after extended testosterone use [40, 41].

Among adolescents, GnRH agonists (GnRHa) may be used to temporarily block puberty and has a reversible effect on follicular maturation and future ovarian function [4, 42]. However, for oocyte cryopreservation, maturation typically requires discontinuing GnRHa to improve ovarian response to stimulation. A case study has documented successful ovarian stimulation, while a patient was still using puberty blockers, suggesting that in some instances, stimulation may be possible without interruption of GnRHa [43].

## Opportunities

### Trans health awareness

Until recently, several European countries required TGD individuals to undergo sterilizing procedures before they could change their legal sex on official documents [44, 45]. This policy was based on the outdated belief that biological parenthood was incompatible with gender affirmation. In 2017, the European Court of Human Rights ruled this practice illegal, marking a significant step forward in in the rights of TGD individuals. [34, 46].

Since then, policies have evolved, and healthcare professionals have become increasingly informed about gender-diverse care. Advocacy from LGBTIQ + communities has played a key role in driving these changes, leading to the establishment of new guidelines that standardize care and improve health outcomes for TGD individuals.

## Diversification of parenthood options

A study of adolescent and young adult cancer survivors found that individuals identifying as LGBTQ were less concerned about how infertility might affect their future romantic relationships and were more open to alternative family structures, such as raising non-biological children or not having children at all compared to their non-LGBT peers [47, 48]. Among TGD youth, a survey showed that even though half wanted to become parents, only a minority valued biological filiation [49]. Adoption was highlighted as an alternative to biological children by those who did not choose to undergo fertility preservation [50, 51]. This option may be particularly appealing to individuals who are unwilling to undergo these treatments and who may find the resulting bodily changes more challenging.

### Advances in assisted reproductive technologies

Advances in ART have reduced barriers to reproduction, expanding access to parenthood for TGD individuals. FP, such as oocyte cryopreservation, is now a feasible option due to improved vitrification techniques, which have significantly enhanced gamete survival rates [52, 53]. Recent studies suggest that pregnancy rates are comparable between fresh and vitrified oocytes, underscoring the effectiveness of these advancements [53].

Ovarian tissue cryopreservation (OTC) may be considered when ovarian stimulation is not a viable option [54]. Live births have been reported among cis women who have undergone OTC, providing some evidence of its potential success [55]. Among individuals undergoing GAT, this method can be combined with GAS and may serve as the only fertility preservation option for individuals before puberty [56]. However, it is important to note that OTC often requires re-transplantation of the ovarian tissue, which may come with potential side effects [57]. According to ASRM guidelines, OTC is no longer considered experimental for post-pubertal individuals [58, 59], though it remains experimental for prepubertal individuals in both ASRM and ESHRE guidelines [60, 61]. Nevertheless, it presents a promising alternative for those unable to undergo traditional ovarian stimulation.

Another promising technique, in vitro maturation (IVM), involves collecting immature oocytes from antral follicles, maturing them under laboratory conditions. Although oocyte IVM is no longer considered experimental in reproductive medicine, it is not available in all fertility treatment laboratories. The results in terms of fertilization are lower compared to those of the oocytes collected after conventional controlled hyperstimulation, but important improvements in the technique have been achieved [62, 63]. IVM has shown potential in TGD individuals, with successful collection of cumulus-oocyte-complexes even under testosterone treatment, though the efficiency remains low [65, 66]. The lack of recommendations for application of this specific technique to fertility preservation in TGD individuals makes it difficult to define a non-experimental setting for use in such cases.

## Weakness

### Dysphoria

Managing hormonal treatments during fertility preservation can be particularly challenging for individuals experiencing gender dysphoria. In those undergoing controlled ovarian stimulation, a washout period from testosterone therapy may heighten distress [24, 67]. The resulting surge in estradiol—along with the potential return of menstruation—can further exacerbate dysphoria, intensifying symptoms that conflict with the individual’s gender identity.

The process also requires frequent ultrasounds, which may increase physical discomfort and trigger gender dysphoria. Additionally, the oocyte retrieval procedure is invasive, involving transvaginal ultrasound-guided needle aspiration of the ovaries under sedation or anesthesia, further adding to the physical and emotional burden [17, 69].

Fertility counseling and conversations about reproductive anatomy can exacerbate dysphoria in who experience distress related to their reproductive organs. It is therefore essential that healthcare professionals receive training to navigate these conversations sensitively, acknowledging the psychological and emotional barriers that may impact decision-making regarding FP.

### Challenges of ovarian stimulation

For postpubertal TGD individuals, oocyte cryopreservation after ovarian stimulation is the standard procedure [70, 71]. Overall, cryopreservation allows individuals to defer decisions about parenthood while preserving their potential to become parents in the future.

A major risk associated with ovarian stimulation is ovarian hyperstimulation syndrome (OHSS), especially in younger individuals with high ovarian reserves [72]. Although the use of GnRH agonists to trigger ovulation has been shown to greatly reduce the risk of OHSS with no negative impact on oocyte quality or quantity [73], the risk of OHSS remains a concern for some patients. Nevertheless, since TGD individuals are often younger than cisgender patients undergoing ovarian stimulation for IVF or elective oocyte vitrification, they tend to have higher numbers of mature oocytes and lower rates of aneuploidy, which can improve the success rates of future ART procedures [74].

### Timing and transition management

Timing and transition management present significant challenges when discussing FP with TGD individuals, especially during adolescence. In the general population, the desire to have children tends to increase with age peaking between 26 and 36 years old [75]. Parenthood is rarely a priority during adolescence, and aside from young patients undergoing gonadotoxic treatments, concerns about fertility are uncommon. As a result, making decisions about future parenthood during this early stage can be particularly challenging [76].

Additionally, some TGD individuals choose not to undergo FP to avoid delaying their transition or due to concerns about temporarily pausing hormone therapy [17, 22]. However, with timely referrals and well-coordinated consultations, delays can be minimized. This underscores the need for proactive management to minimize treatment delays while preserving fertility options.

### Challenges of family-building

Pregnancy following gamete cryopreservation is not guaranteed. With oocyte cryopreservation in particular, the likelihood of a live birth decreases based on the age at which the retrieval was performed, and the number of oocytes collected [74, 77, 78].

For TGD individuals who wish to have biological children, the choice of a partner plays a significant role in shaping their family planning. Depending on the reproductive capabilities of both partners, different paths may be pursued, such as the use of ART, including sperm or egg donation, surrogacy, or reciprocal IVF. However, access to these options remains limited, notably due to high costs and legal restrictions.

Some individuals have never considered their future parenthood plans, whether due to young age, absence of a partner, or an unstable personal situation often compounded by gender dysphoria. Introducing fertility counseling in this context can lead to additional psychological burden [80, 81].

## Strength

### Fertility counseling and discussion about parenthood options

Access to counseling services can help individuals explore their attitudes toward fertility and parenthood while providing support in a shared decision process. Furthermore, fertility counselling is also an opportunity to explore and explain alternative options, such as IVF, surrogacy, gamete donation, and spontaneous conceptions.

### Adapted stimulation protocols

Several adjustments to standard ovarian stimulation protocols can be suggested to meet the needs of TGD individuals. A precautionary 3-month washout from testosterone treatment is typically recommended before starting ovarian stimulation, due to the lack of data on the impact of GAHT on oocyte quality [34, 82, 83]. It has been suggested that testosterone may be continued until and even during ovarian stimulation, following appropriate individual discussions and shared decision-making [57, 82, 84, 86]. The outcomes of stimulation with or without a washout are unclear and may depend on the duration of GAHT. The use of aromatase inhibitors may help decrease estradiol levels while still supporting adequate follicular growth, which could be beneficial in minimizing dysphoria by reducing both circulating estrogen and the intensity of menstrual bleeding [87, 88]. These inhibitors are already widely used in breast cancer patients without compromising stimulation outcomes [90]. Additionally, the levonorgestrel intrauterine hormonal system, often used for birth control or to reduce menstruation, can be left in place during ovarian stimulation without negatively affecting outcomes [73]. Progestin-primed ovarian stimulation is another viable option, using progestin both to prevent premature LH surges and to reduce endometrial thickening, thereby potentially mitigating some of the gender dysphoria caused by stimulation [91]. While these improvements have primarily been developed with cisgender patients in mind, they could also offer significant benefits for TGD individuals undergoing fertility preservation.

To minimize dysphoria related to transvaginal procedures like ultrasound or oocyte retrieval, alternative options exist such as trans abdominal or transrectal ultrasound, and transabdominal egg retrieval. These procedures have been shown to be safe and effective [92, 93] but should be done under sufficient sedation to ensure comfort and reduce dysphoria in those who do not tolerate vaginal procedures.

## Discussion

### Regret

Among individuals who underwent sterilizing procedures before FP options became available, 44% of those assigned female at birth expressed a desire for FP—nine or more years after GAS. The desire for children increased from 34% in adolescence to 56% in adulthood, illustrating that the wish to have children often strengthens over time [94].

Among cancer survivors, it has been shown that iatrogenic infertility can lead to regret and mental distress, especially for pediatric patients [95, 96]. Although the circumstances of cancer patients and TGD individuals differ, both groups exhibit similar levels of regret when FP is not pursued, particularly when reproductive counseling is absent [17, 95–98]. Comprehensive and informed discussions about FP are essential during the transition process to help individuals make informed decisions, prevent potential regret, and address the mental health implications of irreversible steps.

### Gamete storage

Oocyte cryopreservation is usually used as a kind of “fertility insurance” and many of the individuals—both cis and trans—who stored their gametes may never use them in the future. Studies conducted on cancer survivors highlighted that only 6% of cis women used their stored gametes [100]. These data raise important questions about the potential overuse of medical resources and the ultimate disposition of stored oocytes. While they may be used for reproduction, donated to research, or discarded, the latter remains the most common outcome [101]. As the interest in gamete storage grows, concerns about the resources needed for storage, transportation, and their ecological impact are increasing. Offsite storage and the relocation of gametes for reasons such as seeking permissive legislation also add to the environmental burden. Additionally, medical tourism [102] is contributing to ethically questionable practices like commercial surrogacy [103], advanced-aged pregnancies [104], and sex selection [105].

### Need for research

Unfortunately, research on the effects of GAHT on fertility and its reversibility remains limited and is often inconsistent, with contradictions between older and more recent studies [29, 30, 37, 38, 106–108]. This inconsistency is particularly problematic for counseling, as inconclusive data can lead to suboptimal care that is often not adapted to the needs of TGD individuals. Additionally, there is a critical lack of research on the effects of puberty blockers and other gender-affirming interventions on gonads in young adolescents, which is concerning given that decisions about these treatments are often made at the onset of puberty [110]. Longitudinal and prospective studies should be conducted to determine the effects of puberty blockers and GAHT on gonadal function. These treatments present unique challenges for fertility preservation, underscoring the need for further research to develop more effective strategies tailored to this population. Ovarian stimulation protocols have not been standardized, and prospective studies investigating the outcomes of oocyte retrieval and live births for individuals who have undergone oocyte cryopreservation while on testosterone versus those whose testosterone was interrupted would be useful to determine the impact of different washout periods on the success of fertility preservation.

In the absence of accurate, evidence-based guidance, TGD individuals—particularly adolescents—may be compelled to make decisions about their reproductive futures without a full understanding of the potential consequences. Therefore, it is imperative that more research is conducted to inform clinical guidelines and ensure that healthcare providers can offer TGD patients the best possible care.

## Conclusion

In conclusion, it is essential that fertility counseling is offered to all transgender and non-binary individuals seeking GAT. Advances in medical technology and evolving policies now provide the possibility of biological parenthood; however, healthcare professionals must address the unique stigmas and barriers TGD individuals face. They should be informed about the potential effects of hormone therapy on fertility, the cost of procedures, and potential limitations depending on their partner’s reproductive options. Addressing timing and transition management is key for TGD individuals making informed fertility preservation decisions. Balancing current transition goals with future reproductive plans is essential for holistic care. Given the limited data on the reversibility of GAHT and the potential influence of treatment duration, further research is needed. Technological advancements should also focus on prepubertal fertility preservation as more children opt for early transition.

Healthcare providers must consider the medical, psychosocial, and ethical factors influencing fertility decisions, as current fertility counseling is often incomplete and inaccessible, particularly for those from disadvantaged backgrounds [111]. Inclusive practices and policies are urgently needed to improve access to ART. By advocating for policy changes and including TGD voices in research, healthcare systems can create guidelines that respect their reproductive rights, as recognized by the Universal Declaration of Human Rights [112].

## Data Availability

Does not apply as this is a review.
